# Mutation of Glycosylation Sites in BST-2 Leads to Its Accumulation at Intracellular CD63-Positive Vesicles without Affecting Its Antiviral Activity against Multivesicular Body-Targeted HIV-1 and Hepatitis B Virus

**DOI:** 10.3390/v8030062

**Published:** 2016-02-29

**Authors:** Zhu Han, Mingyu Lv, Ying Shi, Jinghua Yu, Junqi Niu, Xiao-Fang Yu, Wenyan Zhang

**Affiliations:** 1 Institute of Virology and AIDS Research, First Hospital of Jilin University, Changchun 130021, China; jully90@163.com (Z.H.); mingyulv@jlu.edu.cn (M.L.); shiy707@nenu.edu.cn (Y.S.); yjh-0-2002@hotmail.com (J.Y.); 2 Department of Hepatology, First Hospital of Jilin University, Changchun 130021, China; niujunqi549b@sina.com (J.N.)

**Keywords:** BST-2, glycosylation, HIV-1, HBV

## Abstract

BST-2/tetherin blocks the release of various enveloped viruses including HIV-1 with a “physical tethering” model. The detailed contribution of N-linked glycosylation to this model is controversial. Here, we confirmed that mutation of glycosylation sites exerted an effect of post-translational mis-trafficking, leading to an accumulation of BST-2 at intracellular CD63-positive vesicles. BST-2 with this phenotype potently inhibited the release of multivesicular body-targeted HIV-1 and hepatitis B virus, without affecting the co-localization of BST-2 with EEA1 and LAMP1. These results suggest that N-linked glycosylation of human BST-2 is dispensable for intracellular virion retention and imply that this recently discovered intracellular tethering function may be evolutionarily distinguished from the canonical antiviral function of BST-2 by tethering nascent virions at the cell surface.

## 1. Introduction

Humans and other mammals are equipped with endogenous cellular defense proteins as host restriction factors to provide resistance to infection, which must be overcome by viruses to facilitate their optimal replication. BST-2/tetherin is such an interferon-inducible antiviral glycoprotein [1,2], consisting of an N-terminal cytoplasmic tail (CT), a transmembrane (TM) domain, a coiled-coil extracellular domain and a glycosyl-phosphatidylinositol (GPI) anchor at the C-terminus [3]. BST-2 inhibits the release of various enveloped viruses [4] by tethering nascent virions at the cell surface, with its GPI anchors incorporated into the virion envelope and TM domains embedded in the host cell membrane to exert antiviral effects [5,6] via a “physical tethering” model that requires its structural domains and specific amino acid sites. HIV-1 Vpu is a 16-kDa type I integral membrane protein [7,8], acting as the viral antagonist of BST-2. BST-2 can be modified by multiple N-linked glycosylations at two conserved asparagine residues in its extracellular domain. Some previous studies have proposed that these residues are important for anti-HIV-1 activity [5,9], while others found that alteration of N-linked glycosylation sites had a negligible effect on virus restriction [10,11,12,13]. Recent studies have shown that mouse and rat BST-2 possess potential residues for glycosylation, but fail to be glycosylated [14]. Therefore, whether N-linked glycosylation plays an essential role in the antiviral activity of BST-2 and its detailed functional contribution remains to be defined. Recent studies have provided the novel finding that BST-2 restricts hepatitis B virus (HBV) at intracellular vesicles including multivesicular bodies (MVBs) [15,16]. Both studies provided evidence to support that BST-2 co-localizes with HBV large surface (LHBs) in MVBs [12]. However, whether N-linked glycosylation is critical for this newly discovered antiviral function is also unknown.

Here, we primarily confirmed that the mutation of glycosylation sites in BST-2 exerted an effect of post-translational mis-trafficking, leading to its accumulation at intracellular CD63-positive vesicles and potently inhibited the release of MVB-targeted HIV-1 and HBV. These results suggest that the recently discovered intracellular tethering function may be evolutionarily distinguished from the canonical antiviral function of BST-2 by tethering nascent virions at the cell surface. This study has provided new concepts for the current understanding of the host restriction factor BST-2.

## 2. Materials and Methods

### 2.1. Cell Culture and Transfections

HEK293T cells (no. CRL-11268), Huh-7 (no. PTA-4583) were obtained from American Type Culture Collection (Manassas, VA, USA) and maintained in Dulbecco’s high glucose modified Eagle’s medium (DMEM) supplemented with 10% fetal bovine serum (FBS). Plasmid transfections were performed using Lipofectamine 2000 (Invitrogen, Carlsbad, CA, USA).

### 2.2. Plasmids

All of the modified human BST-2 variants were engineered with the use of the QuickChange mutagenesis system (Santa Clara, CA, USA), and sequences were confirmed. The pNL4-3 ΔVpu, BST-2 WT IHA, BST-2 ΔKRK and VR1012 vectors have been described previously [17,18,19,20]. The MVB-targeted pNL4-3 ΔVpu MA 29/31KE construct was cloned as described in a previous study [21]. The pCMV ayw HBV proviral construct and LHBs-Flag plasmid were previously described [15,16]. 293T cell lines transduced by BST-2 variants were established by transfection of pLVX-puro-BST-2 WT, BST-2 N65A, BST-2 N92A, BST-2 N65/92A and lentiviral packaging vectors of the Lenti-X HTX packaging system (Clontech, Mountain View, CA, USA).

### 2.3. Antibodies and Reagents

The following antibodies and reagents were used: anti-tubulin mouse monoclonal antibody (mAb), anti-HA mouse mAb and anti-Flag mouse mAb (Covance, Princeton, NJ, USA); anti-BST-2 rabbit mAb (Abcam, Taipei, Taiwan); anti-p24 mouse mAb obtained from an HIV-1 p24 hybridoma (NIH-ARRRP, Carlsbad, CA, USA); alkaline phosphatase-conjugated goat anti-rabbit and anti-mouse IgG secondary antibodies (Jackson, West Grove, PA, USA); anti-EEA1 antibody and anti-LAMP1 antibody (Abcam, Taipei, Taiwan); ER-Tracker Red, PE-conjugated mouse anti-CD63 mAb (clone CLB-gran/12), Alexa Fluor 488 anti-mouse IgG and Alexa Fluor 594 anti-rabbit secondary antibodies (Invitrogen).

### 2.4. Western Blotting

Proteins of cells lysed in RIPA buffer, followed by addition of sample buffer and boiled for 10 min, were separated by SDS-PAGE and transferred onto nitrocellulose membranes. After blocking in non-fat milk, the membranes were probed with various primary antibodies. Secondary antibodies were then used, and staining was carried out with 5-bromo-4-chloro-3indolyl phosphate (BCIP) and nitro blue tetrazolium (NBT) solutions. The blots were quantified using Glyko® Bandscan software 4.0 [22].

### 2.5. Cellular Fractionation

Stably BST-2-expressing 293T cells were mixed with PBS buffer and treated with gentle ultrasonic disruption. The whole cell lysate was added to a sucrose gradient for isolation. The sucrose layer was prepared in a centrifuge tube with 1 mL volumes of 20%, 30%, 40%, 50% and 60% sucrose in PBS. The gradients were spun at 35,000 rpm for 16 h at 4 °C. Eleven 0.5 mL fractions including the upper sample were collected from the top of the gradient.

### 2.6. Immunofluorescence Analysis

Stable 293T cells expressing BST-2 or its variants, or 293T cells seeded on coverslips were transfected with indicated plasmids. After 48 h, cells were fixed with 4% paraformaldehyde and permeabilized with 0.1% Triton X-100, blocked in 10% FBS in PBS and then incubated with anti-BST-2 or anti-HA mAb for 1 h. Cells were then stained with Alexa Fluor 488 goat anti-mouse IgG along with 1 mg/mL DAPI (4,6-diamidino-2-phenylindole) for 1 h. After subsequently washing the cells three times in PBS, the ER was stained for 30 min at 37 °C with ER-Tracker Red, and CD63 was labeled for 1 h at 37 °C with a PE-conjugated mouse anti-CD63 mAb. For the LHBs-Flag test, anti-Flag mAb/Alexa Fluor 488 goat anti-mouse IgG were used for staining LHBs, and anti-BST-2 rabbit mAb/Alexa Fluor 594 goat anti-rabbit IgG for BST-2. For the EEA1/LAMP1 test, anti-HA mAb/Alexa Fluor 488 goat anti-mouse IgG were used for BST-2, and anti-EEA1/LAMP1 rabbit pAb/Alexa Fluor 594 goat anti-rabbit IgG for EEA1/LAMP1. The samples were analyzed on an Olympus IX71 fluorescence microscope. The level of co-localization was quantified by converting RGB images to grayscale images, and the co-localization coefficient (R) in overlapping images was obtained using Image-Pro Plus 6.0 (Media Cybernetics, Rockville, MD, USA).

### 2.7. HIV-1 Production

HIV-1 particles were produced by transient transfection with a proviral construct. After 48 h, cultured supernatants were ultracentrifuged to concentrate the virion particles. Virus particle pellets and corresponding cell lysates were analyzed by SDS-PAGE and Western blotting using an anti-p24 capsid antibody.

### 2.8. HBV Production and Detection

HBV particles were produced in the indicated cells in a 6-well plate with 1 μg HBV proviral construct and the indicated amounts of other plasmids. The cultured medium and cell lysates were examined for HBV surface antigen (HBsAg) and HBV e antigen (HBeAg) with ELISA kits (Kehua, Shanghai, China) according to the manufacturer’s instructions. The microplate was imaged with a scanner (Hewlett-Packard, Palo Alto, CA, USA) and quantified using a microplate reader (Bio-Rad, Hercules, CA, USA). HBsAg in the supernatant was normalized to the HBeAg expression level and converted into percentages.

### 2.9. Statistical Analysis

All statistical data are presented as the mean ± SEM. Statistical significance of the differences was determined using Student‘s *t*-test. Differences were considered significant at values of *p* < 0.05.

## 3. Results

### 3.1. BST-2 with Mutated Glycosylation Sites Translocate to Subcellular Fractions with Higher Densities

**Figure 1:**
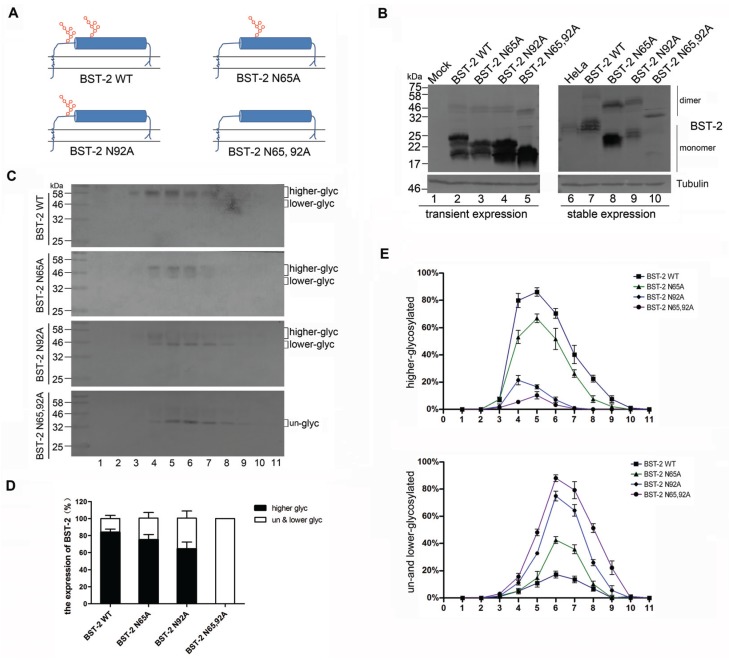
N-linked glycosylation affects subcellular distribution of BST-2. (**A**) Schematic representation of BST-2 variants. Glycosylation sites N65 and N92 are marked in red; (**B**) Comparison of post-translational modifications of transiently- and stably-expressed BST-2 variants. 293T cells were transfected with 200 ng of BST-2 variants. After 48 h, these cells and stably-transduced cells were analyzed by Western blotting using an anti-BST-2 mAb; (**C**) 293T cells stably expressing BST-2 variants in 10-cm dishes were lysed and analyzed by sucrose gradient ultracentrifugation. Samples were analyzed by Western blotting with an anti-BST-2 mAb; (**D**) Percentages shown in black and white columns, respectively, represented the levels of higher-glycosylated or lower-glycosylated and un-glycosylated BST-2 in panel C from three experiments. Levels of glycosylated patterns of BST-2 were quantified by the image J software Results were shown as mean ± SD; (**E**) Levels of glycosylated patterns of BST-2 in each sample in panel C from three experiments were quantified and plotted. Results were shown as mean ± SD. These experiments were repeated three times, and the most representative Western blot images are shown.

N-linked glycosylation of BST-2 plays an important role in the restriction of HIV-1 release from cells and activation of NF-κB signaling [5,9]. To better understand the functional significance of BST-2 glycosylation, asparagines 65 and 92 of human BST-2 were mutated to alanine yielding mutants N65A, N92A and N65/92A (Figure 1A). We analyzed the mobility of BST-2 variants expressed in stably transduced 293T cells and transiently transfected 293T cells by Western blotting. Endogenous BST-2 in HeLa cells appeared as a smear of multiple bands with molecular weight (MW) of about 30 kDa, presumably due to N-linked glycosylation (Figure 1B, lane 6). The transiently-expressed BST-2 in 293T cells exhibited faster mobility than the endogenous protein (Figure 1B, lanes 2–5). Stably-expressed wild-type (WT) BST-2 in 293T cells migrated similarly to the endogenous BST-2 in HeLa cells (Figure 1B, lanes 6 and 7), while glycosylation site mutations (N65A, N92A and N65/92A) reduced the MW of BST-2 (Figure 1B, lanes 3–5 and 8–10).

To investigate the effect of glycosylation on the subcellular distribution of BST-2, we analyzed BST-2 variants with a subcellular fractionation assay. The 293T cells stably expressing BST-2 variants were lysed with moderate sonication to maintain subcellular structures. The lysates were isolated on a sucrose layer with increased densities by ultracentrifugation. Eleven fractions were collected from the top of the gradient and analyzed for BST-2 by Western blotting. BST-2 N65/92A and BST-2 N92A were detected as un-glycosylated and lower-glycosylated forms compared with WT BST-2 and were found mainly in fractions with larger densities (Figure 1C,E). By contrast, BST-2 N65A only exhibited a moderate alteration in localization in the density gradient. In order to further test whether WT BST-2 and variants differ in their subcellular localization, we quantified the percentages of higher-glycosylated or lower-glycosylated and un-glycosylated BST-2 for each BST-2 group. BST-2 N92A, especially BST-2 N65/92A exhibited mainly as un-glycosylated and lower-glycosylated forms compared with WT BST-2 (Figure 1D). These results suggested that the BST-2 proteins with mutated glycosylation sites would exhibit as lower-glycosylated forms and translocate to subcellular fractions with larger densities, which may be vesicular compartments other than the plasma membrane.

### 3.2. BST-2 Proteins with Mutated Glycosylation Sites Accumulate at Intracellular CD63-Positive Vesicles

**Figure 2:**
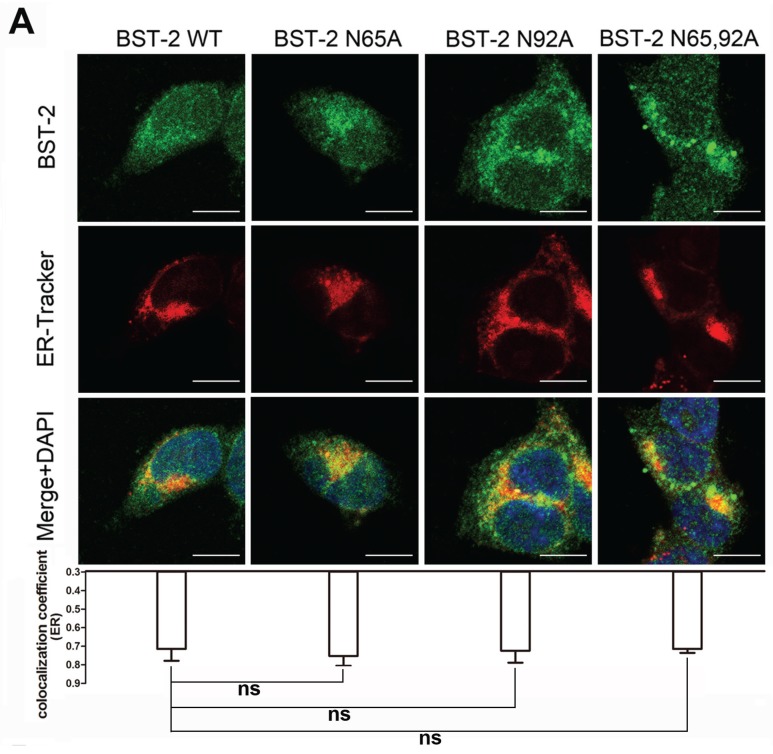

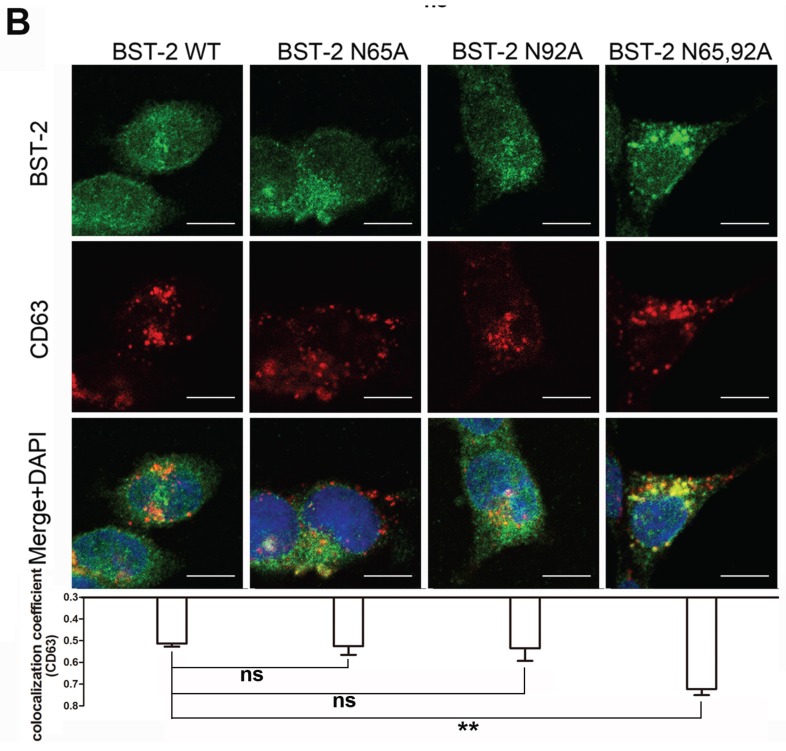
Impairment of N-linked glycosylation induces enhanced subcellular distribution of BST-2 in CD63-positive vesicles. (**A**) 293T cells stably expressing BST-2 or its variants were stained; blue, cell nucleus; green, BST-2 protein; red, ER-Tracker; (**B**) 293T cells stably expressing BST-2 or its variants were stained; blue, cell nucleus; green, BST-2 protein; red, CD63-PE. Images were taken under a Zeiss LZM710 confocal microscope. At least 30 individual cells were examined in each sample, and the most representative cells are shown. The co-localization coefficient (R) was calculated using Image-Pro Plus 6.0. Results were shown as mean ± SD. Statistical comparisons between the groups were performed by unpaired *t*-test using GraphPad Prism 5. ** *p* < 0.01, ns (not significant) *p* > 0.05. Scale bars = 10 μm.

HIV-1 viral particles assemble at different sites in different subtypes of host cells [23]. The majority of virus particles assemble at the cell surface in T cells and several non-hematopoietic cell lines, while in macrophages these events occur almost entirely in intracellular membranes which represent a subset of CD63-positive vesicles [24]. Given the above results, glycosylation possibly can be considered to affect the intracellular localization as well as the subcellular distribution, such as the ER, or CD63-positive vesicles. To confirm this hypothesis, 293T cells stably expressing BST-2 or its variants were used to detect the co-localization with ER-Trackter and CD63. As shown in Figure 2A, most BST-2 variants exhibited a puncta-like distribution. WT BST-2 partly appeared in the ER, and BST-2 glycosylation mutants showed similar profiles. In contrast, BST-2 N65/92A was detected as larger puncta, which co-localized with the CD63-positive compartments (Figure 2B). The results indicated that the BST-2 mutants lacking glycosylation sites were still able to traffic through the ER membrane but then accumulated at the intracellular CD63-positive vesicles.

### 3.3. BST-2 Proteins with Mutated Glycosylation Sites Potently Inhibits MVB-Targeted HIV-1

**Figure 3:**
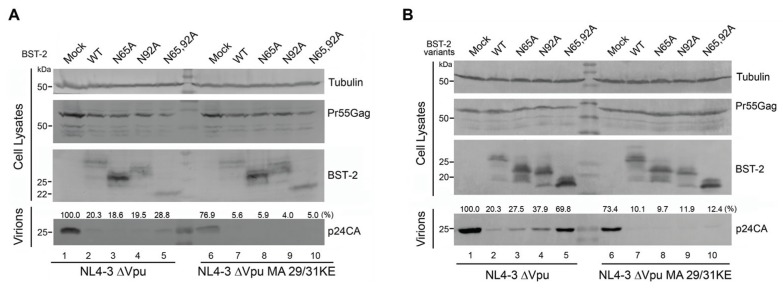
N-linked glycosylation of human BST-2 is dispensable for intracellular MVB-targeted HIV-1 inhibition. (**A**) 293T cells stably expressing BST-2 or its variants in six-well plates were transfected with 1 μg of pNL4-3 ΔVpu or pNL4-3 ΔVpu MA 29/31KE proviral plasmids. After 48 h, cultured supernatants were ultracentrifuged to concentrate the virion particles. Virions and cell lysates were analyzed by Western blotting to detect viral p24CA and intracellular Pr55Gag proteins; (**B**) 293T cells in six-well plates were transfected with 1 μg of pNL4-3 ΔVpu or pNL4-3 ΔVpu MA 29/31KE proviral plasmids and 50 ng of BST-2 variants. After 48 h, cultured supernatants were ultracentrifuged to concentrate the virion particles. Virions and cell lysates were analyzed by Western blotting to detect viral p24CA and intracellular Pr55Gag proteins.

Multiple lines of evidence have indicated that Gag trafficking to CD63-positive compartments, including late endosomes [24] and MVBs [23], occurs prior to viral particle budding from the plasma membrane. The above observations revealed that BST-2 proteins with mutated glycosylation sites accumulate at intracellular CD63-positive vesicles, implying that un-glycosylated BST-2 may exhibit a stronger antiviral activity against viruses that assemble at such locations. To evaluate the intracellular virion tethering function of BST-2 with impaired glycosylation, an MVB-targeted Gag matrix mutation MA 29/31KE was introduced into pNL4-3 ΔVpu to generate pNL4-3 ΔVpu MA 29/31KE. 293T cells stably expressing BST-2 or its variants were transfected with pNL4-3 ΔVpu or pNL4-3 ΔVpu MA 29/31KE. The virion release was examined by evaluating concentrated virions with Western blotting (Figure 3A). The antiviral function of BST-2 in restricting HIV-1 ΔVpu release was attenuated upon the mutation of glycosylation sites (Figure 3A, lanes 3–5). The release of HIV-1 ΔVpu MA 29/31KE was also notably restricted in the presence of WT BST-2 (Figure 3A, lane 7). However, no obvious attenuation in restricting HIV-1 ΔVpu MA 29/31KE release was observed in the presence of any BST-2 variant with mutated glycosylation sites (Figure 3A, lanes 8–10). Similar results were obtained in transfected 293T cells with pNL4-3 ΔVpu or pNL4-3 ΔVpu MA 29/31KE along with indicated BST-2 variant (Figure 3B). This result indicated that the BST-2 mutants lacking glycosylation sites were still able to inhibit the release of HIV-1 viruses that assembled in MVBs.

### 3.4. BST-2 Proteins with Mutated Glycosylation Sites Show Significant Co-Localization with LHBs and Potently Inhibit HBV Release

**Figure 4:**
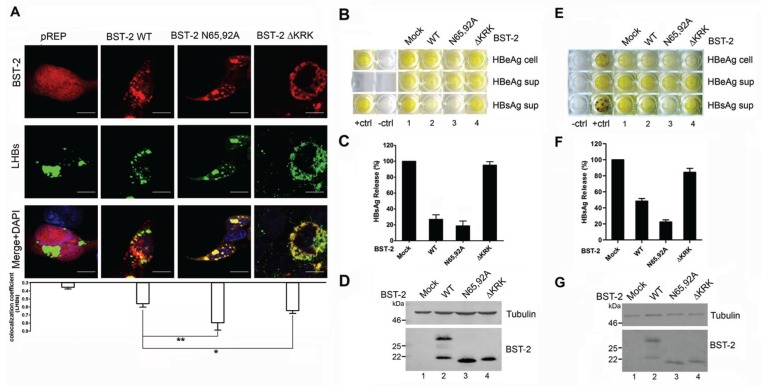
BST-2 with mutated glycosylation sites significantly co-localizes with LHBs and potently inhibits HBV production. (**A**) 293T cells transfected with 1 μg of LHBs-Flag along with 800 ng of BST-2 or its variants or pRFP vectors were stained; blue, cell nucleus; green, LHBs protein; red, BST-2 protein. Images were taken under a Zeiss LZM710 confocal microscope. At least 30 individual cells were examined in each sample, and the most representative cells are shown. The co-localization coefficient (R) was calculated using Image-Pro Plus 6.0. Results were shown as mean ± SD. Statistical comparisons between the groups were performed by unpaired t-test using GraphPad Prism 5. ** *p* < 0.01, * *p* < 0.05. Scale bars = 10 μm; (**B**) 293T cells were co-transfected with 50 ng of BST-2 or its variants along with 1 μg of HBV proviral plasmid. HBV antigens in the cells and supernatants (sup) were detected by HBV antigen ELISA; (**C**) HBsAg release percentages of (**B**) are shown in columns; (**D**) BST-2 expression was detected by Western blotting; (**E**) Huh7 cells were co-transfected with 150 ng of BST-2 or its variants along with 1 μg of HBV proviral plasmid. HBV antigens in the cells and supernatants (sup) were detected by HBV antigen ELISA; (**F**) HBsAg release percentages of (**E**) are shown in columns; (**G**) BST-2 expression was detected by Western blotting.

Recent studies reported that BST-2 restricts HBV, which is a typical virus that assembles at intracellular vesicles including MVBs [15,16]. Both of those studies concerning BST-2-induced HBV restriction provided evidence that BST-2 co-localizes with HBV large surface antigen, which is the major structural protein forming HBV virions. A previously reported BST-2 ΔKRK variant that fails to traffic through the ER membrane leading to glycosylation deficiency was used as a negative control [18]. Here, to confirm whether the intracellular CD63 pattern of BST-2 N65/92A may correlate with LHBs, cellular localization of BST-2 variants and LHBs was first examined in transient transfected 293T cells (Figure 4A). BST-2 N65/92A exhibited an enhanced intracellular punctate pattern and relatively higher co-localization with LHBs compared with the WT BST-2. In contrast, BST-2 ΔKRK exhibited a perinuclear pattern, and no notable co-localization with LHBs was observed. Further analysis confirmed that BST-2 N65/92A exhibited considerable activity against HBV enveloped particles and virions release compared with WT BST-2 (Figure 4B–D). In contrast, BST-2 ΔKRK with a similar glycosylation phenotype failed to inhibit HBV. In order to further confirm above conclusion in hepatocyte cells, we further used Huh7 cells to detect anti-HBV activity of WT BST-2, N65/92A and ΔKRK. The same results were obtained in Huh7 cells (Figure 4E–G). These results confirmed that un-glycosylated BST-2 were still able to inhibit the release of viruses assembled in CD63-positive vesicles in both 293T and hepatocyte cells.

**Figure 5:**
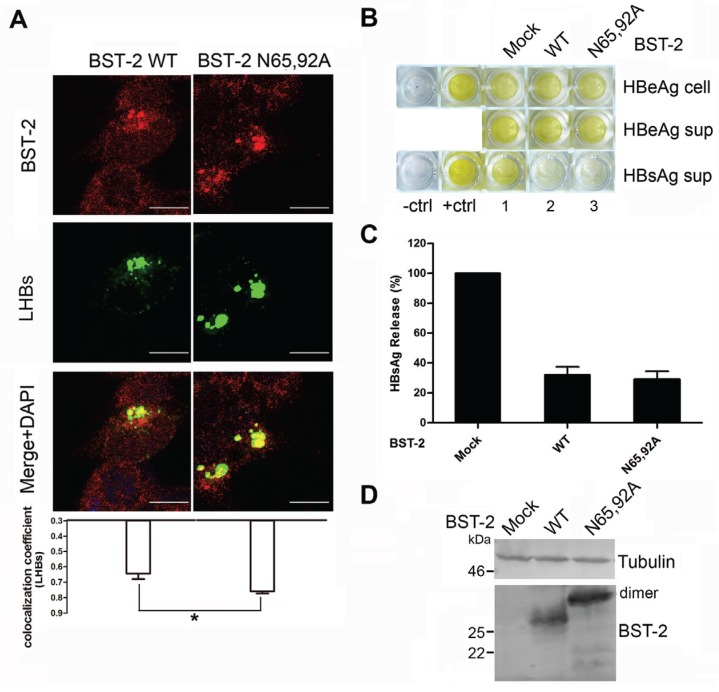
Stably-expressed BST-2 with mutated glycosylation sites significantly co-localizes with LHBs and potently inhibits HBV production. (**A**) 293T cells stably expressing BST-2 or its variants transfected with 1 μg of LHBs-Flag were stained; blue, cell nucleus; green, LHBs protein; red, BST-2 protein. Images were taken under a Zeiss LZM710 confocal microscope. At least 30 individual cells were examined in each sample, and the most representative cells are shown. The co-localization coefficient (R) was calculated using Image-Pro Plus 6.0. Results were shown as mean ± SD. Statistical comparisons between the groups were performed by unpaired *t*-test using GraphPad Prism 5. * *p* < 0.05. Scale bars = 10 μm; (**B**) 293T cells stably expressing BST-2 or its variants were transfected with 1 μg of HBV proviral plasmid. HBV antigens in the cells and supernatants (sup) were detected by HBV antigen ELISA; (**C**) HBsAg release percentages of (**B**) are shown in columns; (**D**) BST-2 expression was detected by Western blotting.

Subsequently, 293T cells stably expressing BST-2 or N65/92A mutant were used to repeat above assays although without BST-2 ΔKRK as a negative control. The result showed that BST-2 N65/92A also exhibited an enhanced intracellular punctate pattern and relatively higher co-localization with LHBs compared with the WT BST-2 (Figure 5A). Thus, although the expression patterns of BST-2 and its variants were different in transient transfected 293T and stable 293T cells (Figure 1B), both of them have similar anti-HBV activity (Figure 5B–D).

### 3.5. Glycosylation of BST-2 Does not Affect Its Co-Localization with EEA1/LAMP1

**Figure 6:**
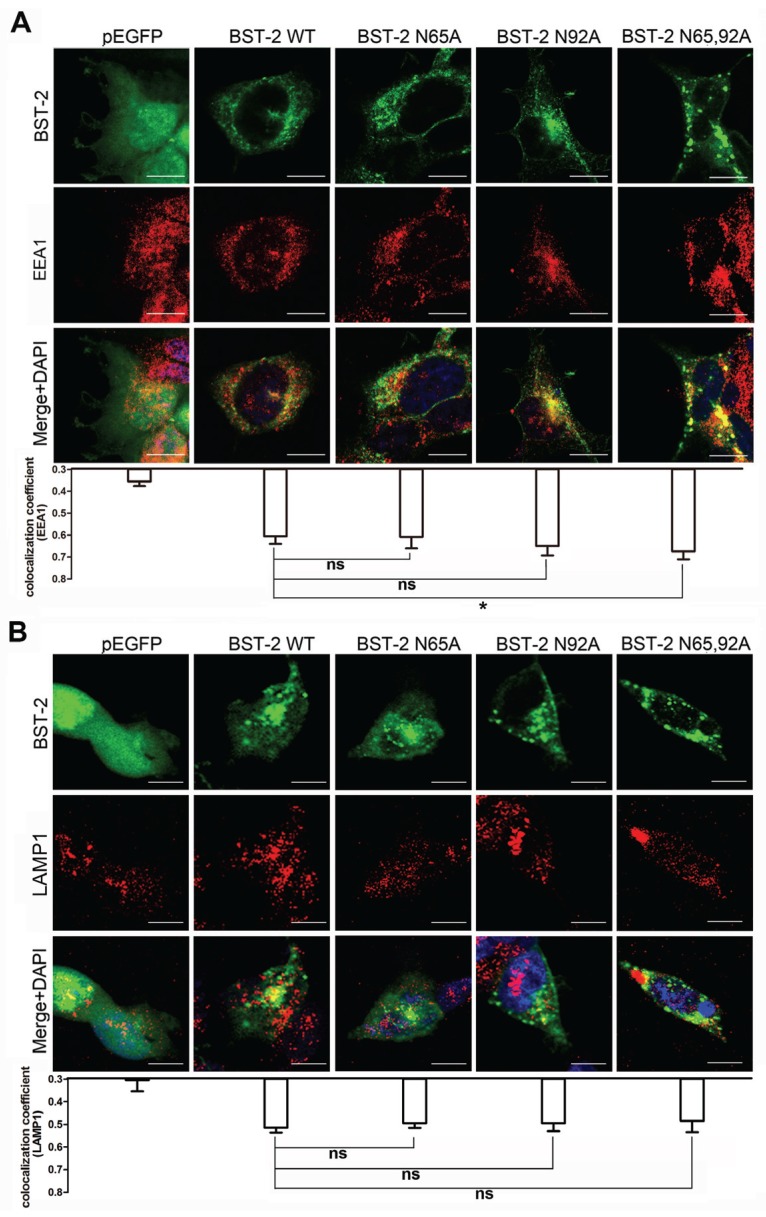
All BST-2 variants partially co-localize with EEA1 and LAMP1. (**A**) 293T cells transfected with 800 ng of BST-2 or its variants expression plasmids or pEGFP vectors were stained; blue, cell nucleus; green, BST-2 protein; red, EEA1 for early endosome; (**B**) 293T cells transfected with 800 ng of BST-2 or its variants expression plasmids or pEGFP vectors were stained; blue, cell nucleus; green, BST-2 protein; red, LAMP1 for lysosome. Images were taken under a Zeiss LZM710 confocal microscope. At least 30 individual cells were examined in each sample, and the most representative cells are shown. Co-localization coefficient (R) was calculated using Image-Pro Plus 6.0. Results were shown as mean ± SD. Statistical comparisons between the groups were performed by unpaired *t*-test using GraphPad Prism 5. * *p* < 0.05, ns (not significant) *p* > 0.05. Scale bars = 10 μm.

We further analyzed the localization using the following endosome markers: EEA1 for early endosomes and Lamp1 for late endosomes/lysosomes. Quantitative analysis of the co-localization indicated no significant difference in the co-localization ratios of WT and mutant BST-2 with EEA1 or LAMP1 in 293T cells except that BST-2 N65,92A has a gently increase with EEA1 (Figure 6). These results indicated that all of the BST-2 mutants could be partially degraded through the lysosomal pathway, and glycosylation had no effect on the degradation pathway.

## 4. Discussion

A “physical tethering” model of BST-2/tetherin has been proposed and several functional roles of its structural domains and sites have been defined. However, the mechanism for the contribution of N-linked glycosylation to the antiviral model is not yet clear. N-linked glycosylation sites of BST-2 were previously shown to affect its expression at the cell surface [5]. In this study, we confirmed that the mutation of glycosylation sites caused more accumulation of BST-2 at intracellular CD63-positive vesicles than WT BST-2 due to its post-translational trafficking, indicating that BST-2 with this phenotype potently inhibited the release of multivesicular body-targeted HIV-1 and HBV. These results suggest that N-linked glycosylation of human BST-2 is necessary for its canonical antiviral function but dispensable for intracellular virion retention.

The low-glycosylated forms of BST-2 were heavily overexpressed relative to mature BST-2 in transiently-transfected 293T cells. The stably-expressed BST-2 exhibited similarities with that of the endogenous protein, although multiple glycosylation forms were present (Figure 1B). The transiently-expressed BST-2 were excessively glycosylated or bypassed the glycosylation machinery in the Golgi. Although the stably-expressed BST-2 appeared to have fewer glycosylated forms, multiple bands could still be observed by SDS-PAGE. These multiple forms could be isolated by the cellular fractionation (Figure 1C). The highly and lowly glycosylated forms showed an unbalanced distribution in the subcellular regions with different densities. The mutation of glycosylation sites resulted in more abundant lower-glycosylated of BST-2 proteins, which were found in fractions with larger densities compared with the higher-glycosylated form.

The above phenotype could be attributed to the mis-folding of the protein and its retention in ER membranes. However, the protein alternatively may have undergone unsuccessful trafficking to plasma membranes and as a result was retained in certain vesicular compartments. Thus, further investigations were performed using immunofluorescence assays. The former possibility was excluded by evaluating the co-localization of BST-2 variants via ER staining, which exhibited a typical perinuclear pattern (Figure 2A). Although BST-2 showed moderate co-localization with the ER, which is characteristic of newly synthesized proteins, immunoflurourescence detection of both WT BST-2 and glycosylation mutants showed considerable amounts of punctated signals in the outer cytosol, suggesting that they were properly folded and efficiently detached from the ER. CD63 is a vesicular marker which represents the typical trafficking route of various membrane proteins. The site of HIV assembly in human macrophages where BST-2 tethers virions to virus-containing vesicles had been identified as CD63-positive multivesicular bodies [25]. The latter possibility mentioned above was examined by the co-localization analysis of BST-2 variants with CD63. BST-2 N65/92A and BST-2 N92A both exhibited as larger puncta, which accumulated in CD63-positive compartments (Figure 2B). These observations indicate that glycosylation maintains effective intracellular trafficking of BST-2 to the plasma membrane, minimizing the abnormal accumulation in the trafficking route.

Targeting and assembly of Gag in the MVB have been shown to be physiologically important steps in HIV-1 virus particle production in macrophages, and particle release in this cell type may follow an exosomal pathway [23]. We hypothesized that un-glycosylated BST-2 may exhibit stronger antiviral activity against viruses that assemble in a subset of CD63-positive compartments than those viruses released at the cell surface. This hypothesis was primarily confirmed by the antiviral experiment against an MVB-targeted HIV-1 variant, in which MVB-targeted HIV-1 release was potently inhibited by BST-2 glysosylation mutants (Figure 3). As a recently discovered target of the spectrum of BST-2 antiviral activities, HBV displays a typical pattern of assembly in intracellular vesicles [15,16]. The BST-2 N65/92A mutant exhibited even larger puncta compared with WT BST-2 and highly co-localized with LHBs, the major structural component of HBV particles (Figure 4A and Figure 5A). The results demonstrated the potent antiviral activity of the BST-2 glycosylation mutants against HBV release.

Previous studies proposed two degradation pathways to account for the Vpu-induced BST-2 downregulation, the proteasomal degradation [14,26] and lysosomal [27,28] pathways. Here, we analyzed the localization of WT BST-2 and glycosylation mutants with EEA1 or LAMP1 (Figure 6). Both WT BST-2 and glycosylation mutants exhibited considerable co-localization ratios, implying that the mutation of glycosylation sites affected only the localization in the CD63-positive compartment, without affecting the degradation pathway.

Ongoing research has challenged the previous notion that N-linked glycosylation of BST-2 is entirely essential for its antiviral activity. For example, a recent study reported that the antiviral function of feline BST-2 is independent of its N-linked glycosylation [13]. A balance could be controlled through regulating BST-2 glycosylation by related cellular machinery to maintain an effective inhibition of virion production from intracellular vesicles and the plasma membrane. This concept deserves further efforts to clarify whether the functional regulation of BST-2 glycosylation was gained during evolution, as well as the potential correlation between the glycosylation and those viral antagonisms without the surface removal of BST-2. Such studies may provide more insights into the molecular antiviral mechanism of BST-2/tetherin.

## 5. Conclusions

In this study, we primarily confirmed that the mutation of glycosylation sites in BST-2 exerted an effect of post-translational mis-trafficking, leading to its accumulation at intracellular CD63-positive vesicles. BST-2 with this phenotype potently inhibited the release of MVB-targeted HIV-1 and HBV. Additionally, BST-2 with mutated glycosylation sites but not BST-2 with impaired trans-endoplasmic reticulum (ER) ability showed significant co-localization with LHBs. However, the mutation of glycosylation sites had no effect on the co-localization of BST-2 with EEA1 and LAMP1. These results suggest that the recently discovered intracellular tethering function may be evolutionarily distinguished from the canonical antiviral function of BST-2 by tethering nascent virions at the cell surface. This study has provided new concepts for the current understanding of the host restriction factor BST-2.
